# A flow resistive inspiratory muscle training mask worn during high-intensity interval training does not improve 5 km running time-trial performance

**DOI:** 10.1007/s00421-020-04505-3

**Published:** 2020-10-01

**Authors:** Mark A. Faghy, Peter I. Brown, Nicola M. Davis, J. P. Mayes, Tom M. Maden-Wilkinson

**Affiliations:** 1 Human Science Research Centre, University of Derby, Kedleston Road, Derby, DE22 1GB UK; 2 English Institute of Sport, EIS, Sports Training Village, University of Bath, Bath, UK; 3 School of Allied Health and Social Care, University of Derby, Derby, UK; 4 Physical Activity, Public Health and Wellness Research Group, Sheffield Hallam University, Sheffield, UK

**Keywords:** Running performance, High-intensity interval training, Inspiratory muscle training, Flow resistive face masks

## Abstract

**Purpose:**

There is little evidence of the ergogenic effect of flow-resistive masks worn during exercise. We compared a flow-resistive face mask (MASK) worn during high-intensity interval training (HIIT) against pressure threshold loading inspiratory muscle training (IMT).

**Methods:**

23 participants (13 males) completed a 5 km time trial and six weeks of HIIT (3 sessions weekly). HIIT (*n* = 8) consisted of repeated work (2 min) at the speed equivalent to 95% ${\dot{\text{V}}}$O_2_ peak with equal rest. Repetitions were incremental (six in weeks 1, 2 and 6, eight in weeks 3 and 4 and ten in week 5). Participants were allocated to one of three training groups. MASK (*n* = 8) wore a flow-resistive mask during all sessions. The IMT group (*n* = 8) completed 2 × 30 breaths daily at 50% maximum inspiratory pressure (*P*_Imax_). A control group (CON, *n* = 7) completed HIIT only. Following HIIT, participants completed two 5 km time trials, the first matched identically to pre-intervention trial (ISO time), and a self-paced effort.

**Results:**

Time trial performance was improved in all groups (MASK 3.1 ± 1.7%, IMT, 5.7 ± 1.5% and CON 2.6 ± 1.0%, *p* < 0.05). IMT improved greater than MASK and CON (*p* = 0.004). Post intervention, *P*_Imax_ and diaphragm thickness were improved in IMT only (32% and 9.5%, respectively, *p* = 0.003 and 0.024).

**Conclusion:**

A flow-resistive mask worn during HIIT provides no benefit to 5 km performance when compared to HIIT only. Supplementing HIIT with IMT improves respiratory muscle strength, morphology and performance greater than HIIT alone.

## Introduction

Inspiratory Muscle Training (IMT) increases the strength of the chest wall inspiratory muscles and the diaphragm (Brown et al. 2014) and attenuates exercise-induced inspiratory muscle fatigue (Romer and McConnell 2004) and may improve exercise performance, for a review see HajGhanbari et al. (2013). Inspiratory flow-resistive loading (FRL) is a method of IMT that improves respiratory muscle strength and endurance, leading to increased time to exhaustion in cycling (Enright et al. 2006) and treadmill running (Mickleborough et al. 2010). FRL training at 80% of *P*_Imax_, performed 3 times weekly attenuates minute ventilation ($\dot{\text{V}}$_E_), oxygen consumption ($\dot{\text{V}}$O_2_), heart rate, blood lactate (La-) and perceptual responses during cycling exercise (Gething et al. 2004; Enright et al. 2006).

FRL targets the inspiratory muscles according to Ohms law of resistance. The use of high-force/low-velocity or low-force/high-velocity contractions through a variable size aperture creates a resistive load for the inspiratory muscles (Romer and McConnell 2004). Despite the documented benefits, FRL and other IMT methods have been criticised for poor ecological validity as they fail to reflect dynamic inspiratory muscle function during exercise (McConnell 2013). Recently, there has been a rise in the popularity of affordable FRL devices that can be worn during exercise. These mask-type devices, create a seal around the nose and mouth and require participants to inspire through a variable-sized aperture, providing an inspiratory load to breathing during exercise tasks (Shei et al. 2016). Whilst the stimulus to respiratory and other physiological systems are still to be determined, these approaches are suggested to provide a time-efficient approach to conducting IMT methods during exercise training and addressing previous concerns with ecological validity.

Despite increased popularity, no study has compared these masks with more established forms of IMT. Porcari et al. (2016) observed improved markers of endurance performance (ventilatory threshold, respiratory compensation threshold and power output at both ventilatory threshold and respiratory compensation threshold) when compared to a control group, following 6 weeks of wearing an Elevation training mask during high-intensity cycle ergometer training. However, there were no between-group differences in $\dot{\text{V}}$O_2_peak and peak power output in between the experimental and control group. The authors concluded by stating that the mask did not simulate altitude but rather demonstrated properties (i.e. increased respiratory muscle loading) associated more closely with respiratory muscle training techniques. Further to this, Segizbaeva and Aleksandrova (2018) demonstrated a 12% increase in maximum inspiratory pressure (*P*_Imax_) alongside a 3% and 7% improvement in 100 m and 3000 m running performance when using an FRL mask during a 12-week intervention. This study used the device in conjunction with a 12-week training programme consisting of middle distance and whole-body resistance activities that were conducted twice weekly.

The need for inspiratory muscle training techniques that can be used during exercise conditions and specifically target the ventilatory profiles achieved during exercise could address previous validity concerns and offer increased benefit to performance. Although research in this space has demonstrated improved respiratory muscle function (i.e. strength) and endurance performance characteristics, no study has compared the ergogenic properties against pressure threshold IMT. Accordingly, this study aimed to test the efficacy of an FRL mask worn during interval training sessions against pressure threshold methods to determine the benefits to time-trial running performance, diaphragm thickness and respiratory muscle strength.

## Methods

### Participants

Following ethics, approval from the host University, twenty-three (*M* = 13, *F* = 10, Table 1) healthy, non-smoking and recreationally trained runners (mean indoor 5 km personal best 26.15 ± 3.19 min; range: 20.73–33.17 min) provided informed written consent. All female participants were taking oral contraceptives throughout their involvement in this study. Before each trial, participants were instructed to avoid exercise on the day preceding and the day of each exercise test. Participants abstained from alcohol and caffeine in the 24 h preceding all trials and completed a 24 h diet record before their first preliminary trial which was replicated in subsequent visits.

**Table 1:** Descriptive Characteristics of study participants (*n* = 23)

	Pooled mean	MASK (*n* = 8)	IMT (*n* = 8)	CON (*n* = 7)
Age (years)	36.5 ± 9.6	37.7 ± 11.9	36.5 ± 9.4	35.2 ± 8.5
Height (m)	1.72 ± 0.09	1.77 ± 0.07	1.71 ± 0.11	1.68 ± 0.09
Body Mass (kg)	74.1 ± 14.5	80.3 ± 10.4	68.5 ± 15.0	73.6 ± 17.9
FEV_1_ (L)	3.6 ± 0.4	4.0 ± 0.9	3.5 ± 1.1	3.3 ± 0.9
FVC (L)	4.6 ± 0.4	5.1 ± 0.8	4.5 ± 1.5	4.3 ± 1.0
FEV_1_/FVC (%)	77 ± 2	77 ± 8	78 ± 7	75 ± 6
PEF (Ls)	7.4 ± 0.7	6.9 ± 0.6	8.2 ± 0.9	7.1 ± 0.5
$\dot{\text{V}}$O_2_peak (l min^−1^)	3.5 ± 0.6	3.7 ± 0.6	3.6 ± 0.7	3.3 ± 0.2
$\dot{\text{V}}$O_2_peak (ml kg^−1^ min ^−1^)	50.4 ± 6.5	48.9 ± 7.1	53.5 ± 6.1	50.2 ± 6.6
Training speed (km h)	13.2 ± 1.4	13.3 ± 1.4	13.7 ± 2.0	12.6 ± 0.6
Training compliance (%)	90.3 ± 6.7	88.8 ± 8.6	91.8 ± 4.9	90.2 ± 7.2

Values presented as mean ± SD. Inspiratory Muscle Training (IMT), Control group (CON). Peak oxygen uptake ($\dot{\text{V}}$O_2peak_)

### Experimental design

Participants completed a total of five laboratory visits, which were separated by a 6-week training intervention. During visit 1, participants were familiarised with all experimental methods and completed an incremental $\dot{\text{V}}$O_2_peak test. During visit 2, diaphragm thickness was assessed and participants completed a 5 km treadmill time-trial. Following visit 2, participants were randomly allocated to one of three training groups and completed six weeks of HIIT. Group 1 consisted of HIIT whilst wearing an FRL training mask (MASK, *n* = 8, F = *4*), group 2, completed IMT as an adjunct to HIIT (IMT, *n* = 8, *F* = 4) and group 3, a control group (CON, *n* = 8, *F* = 3) who completed HIIT only. In visit 3, participants were re-familiarised with all experimental measures and completed a 5 km time trial. In visit 4, an ISO trial that was matched identically to visit 2. Following a minimum of seven days’ rest, diaphragm thickness was re-assessed, and participants completed a self-paced time-trial (visit 5) Table 2.

**Table 2:** Physiological and perceptual responses for participants in the IMT group

	Pooled Baseline	IMT (*n* = 8)					
		Pre-intervention TT		Post intervention (ISO-Time)		Post intervention TT	
		2.5 km	5 km	2.5 km	5 km	2.5 km	5 km
Time (min)	–	–	25.97 ± 2.07	–	25.97 ± 2.07	–	24.54 ± 1.76^B^
P_Emax_ (cmH_2_O)	105 ± 10 (100 ± 18%)	–	95 ± 12	–	100 ± 8	–	98 ± 15
La- (mmol·l^−1^)	1.6 ± 0.6	–	6.7 ± 3.3	–	4.8 ± 1.9^B^	–	6.4 ± 3.7
HR (beats·min^−1^)	70 ± 18	156 ± 16	177 ± 16 ^a^	150 ± 13	168 ± 7^a^	153 ± 20	178 ± 11^a^
$\dot{\text{V}}$_E_ (L·min^−1^)	14.4 ± 6.8	77.9 ± 24.1	100.2 ± 30.0 ^a^	74.9 ± 19.0	95.7 ± 24.6^a^	87.7 ± 30.5	104.6 ± 33.8^a^
*V*_T_ (L)	0.7 ± 0.1	2.4 ± 0.3	2.8 ± 0.4 ^a^	2.6 ± 0.0	2.8 ± 0.5^a^	2.6 ± 0.6	2.7 ± 0.1^a^
*f*_B_ (b min^−1^)	20 ± 6	47 ± 3	52 ± 5 ^a^	41 ± 3	48 ± 2 ^a^	43 ± 6	50 ± 10^a^
$\dot{\text{V}}$O_2_ (L·min^−1^)	0.46 ± 0.25	2.61 ± 0.95	2.96 ± 1.09 ^a^	2.68 ± 0.77	2.81 ± 1.04^a^	2.94 ± 1.13	3.26 ± 1.17^a^
$\dot{\text{V}}$CO_2_ (L·min^−1^)	0.33 ± 0.27	2.59 ± 0.99	3.05 ± 1.17 ^a^	2.62 ± 0.73	3.15 ± 0.90^a^	2.87 ± 1.16	3.29 ± 1.26^a^
RER	0.92 ± 0.09	0.99 ± 0.04	1.13 ± 0.11 ^a^	0.98 ± 0.05	1.15 ± 0.11 ^a^	0.97 ± 0.03	1.17 ± 0.04^a^
RPE (AU)	6 ± 0	13 ± 1^a^	15 ± 1 ^a^	12 ± 1	13 ± 1^a^	11 ± 3	15 ± 2^a^
RPE_breathing_ (AU)	0 ± 0	3 ± 1^a^	6 ± 2 ^a^	3 ± 1	5 ± 2^a^	3 ± 1	7 ± 3^a^

Values presented as mean ± SD. Maximum expiratory pressure (*P*_Emax_), values in parenthesis represent percentage of predicted, Heart rate (HR), Blood Lactate (La-), minute ventilation (*V*_E_), tidal volume (*V*_T_), breathing frequency (*f*_B_), oxygen consumption ($\dot{\text{V}}$O_2_), carbon dioxide production $\dot{\text{(V}}$CO_2_), Respiratory exchange ratio (RER), Rating of Perceived Exertion (RPE), Rating of Perceived Exertion For Breathing (RPE_breathing_), Arbitrary units (AU). A denotes change between 2.5 km and 5 km values and B represents the difference between trials (*p* < 0.05)

### Preliminary trials

Peak oxygen uptake ($\dot{\text{V}}$O_2_peak) was determined on a motorised treadmill (Desmo, Woodway, Germany) using a maximal incremental exercise test for the baseline assessment of aerobic fitness. Following a 5 min warm-up at 8 km h^−1^ and 1% gradient, the gradient was subsequently increased to 4% and speed increased by 1 km h^−1^ min^−1^ until the limit of volitional tolerance. Participants wore a Hans Rudolph reusable face mask and online breath by breath gas analysis was conducted (Metalyzer 3B, Cortex Biophysik, Leipzig, Germany). Two-point calibration was completed before each test to ensure the accuracy of the gas (O_2_; chemical fuel cell and CO_2;_ non dispersive infrared) and flow (TripleV) sensors. Gas concentrations and flow characteristics of exhaled breath were measured to determine $\dot{\text{V}}$O_2_peak, defined as the highest rolling 30 s mean $\dot{\text{V}}$O_2_ recorded during the test. Previously published guidelines were used to calculate $\dot{\text{V}}$O_2_peak (Howley et al. 1995). Following a minimum of seven days’ rest after this test, participants completed a familiarisation of the 5 km time-trial which is described in full below.

**Fig. 1:**
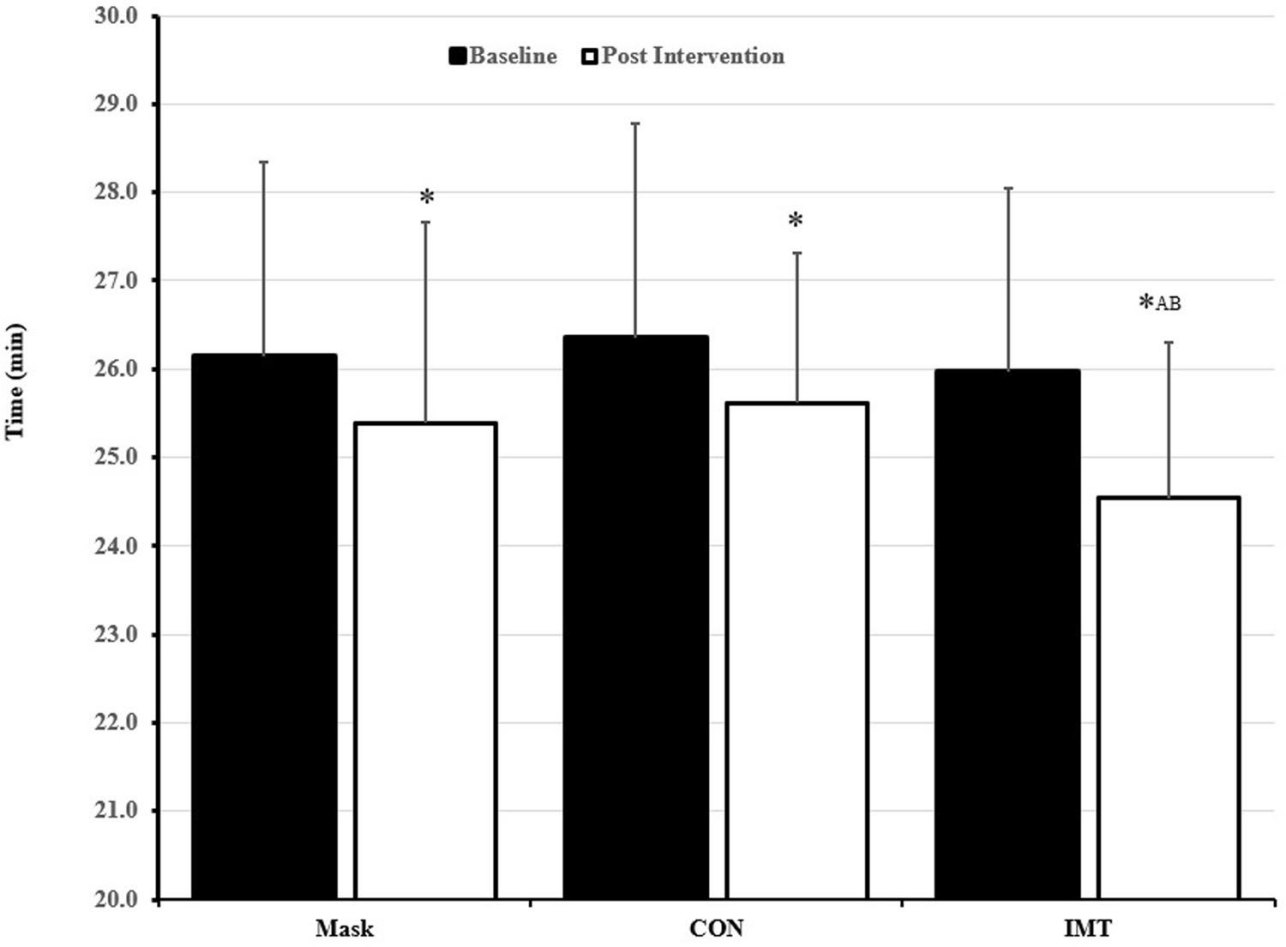
Pre and post-intervention changes in *P*_Imax_ for each condition, values are presented as mean ± std. **a** Different to baseline (*p* < 0.05), *denotes a significant difference baseline

During visit 2, diaphragm thickness (*T*_di_) was assessed via ultrasound (Philips iU22, Guilford, UK) as previously described by our group (Brown et al. 2013). Briefly, the diaphragm was assessed in the zone of apposition with an L17-5 MHz linear array transducer that was adjusted accordingly to the depth of the diaphragm. Participants were positioned upright with their right arm raised and the transducer position was then adjusted between the 7th and 10th intercostal space in the mid-axillary line where a coronal view of the right hemidiaphragm was identified. Fine adjustment of the transducer position was used to place the diaphragm in a horizontal plane across the field of view and to ensure a 90° angle of insonation. Measurements were recorded from functional residual capacity following a passive expiration from total lung capacity. *T*_di_ was defined by onscreen callipers positioned at 90° to the diaphragm from the leading edge of the pleural membrane to the leading edge of the peritoneum membrane (see Fig. 1). Measurements were repeated in triplicate and averaged for subsequent analyses. The within-trial coefficient of variation for *T*_di_ was 2.6% with a within-participant intra-class correlation coefficient of 0.99 which was in accordance with our previous work (Brown et al. 2013).

### Experimental trials

Participants completed a 5 km time trial on a motorised treadmill using the protocol of Driller et al. (2017). Participants were blinded to their elapsed time and standardised verbal encouragement was given (in line with the collection of physiological measures and perceptual responses) throughout the trial. The trial was commenced from a stationary start and running speed was manually adjusted by the participant to complete the distance in the quickest time possible. Changes in speed and the time of adjustment were recorded for the subsequent ISO trial. Prior to exercise, pulmonary function, inspiratory (*P*_Imax_) and expiratory (*P*_Emax_) muscle pressures were measured following published guidelines (American Thoracic Society and European Respiratory Society 2002). Briefly, a hand-held mouth pressure meter (MicroRPM; CareFusion, Hampshire, UK) measured *P*_Imax_ and *P*_Emax_, with manoeuvres initiated from residual volume and total lung capacity, respectively, and sustained for at least 3 s. A minimum of five attempts was recorded with a 30 s rest between each attempt. Dynamic Pulmonary function for the measurement of lung function (FEV_1_, FVC, FEV_1_/FVC, and PEF) was assessed using an electronic flow sensor (MS03, Micro Medical, Buckinghamshire, UK). Respiratory mouth pressures and then pulmonary function were assessed immediately post exercise.

Blood lactate concentration (La-) was measured pre- and post-trial using fingertip capillary samples (Biosen, EKF Diagnostics, Barleben, Denmark). All other parameters including heart rate (Polar T31, Kempele, Finland) and perceptual responses including whole body perceived effort (Borg 1982) and breathing discomfort (using a visual analogue scale: where 0 = no exertion and 10 = maximal exertion, Verges et al. 2007) were measured, upon completion of each kilometre and during the final 50 m of the time-trial. Expired pulmonary gases (O_2_, and CO_2_) and flow characteristics (breathing frequency, tidal volume, and minute ventilation) were measured continuously throughout the trial (averaged over 30 s during analysis) using online gas analysis (Metalyzer II; Cortex Biophysik, Leipzig, Germany).

Following training and re-familiarisation, *T*_di_ was re-assessed and participants completed two additional experimental 5 km time trials. The first was an ISO trial which was matched identically to the speed recorded during the pre-intervention trial. Changes in treadmill speed for the ISO trial were automated using Metasoft® Studio (Cortex Biophysik, Leipzig, Germany). Following a minimum of seven days, a second self-paced, all-out time trial was completed. Training activities were reduced to a maintenance load for all participants between experimental trials and consisted of two HIIT sessions (6 repetitions), IMT was also reduced to a maintenance load (3 × sessions weekly) following previous recommendations (Romer and McConnell 2003).

### Training interventions

Participants were randomly allocated to one of the three training groups: inspiratory FRL training using a mask (MASK), pressure threshold Inspiratory Muscle Training (IMT) or a control group. Participants completed six weeks of high-intensity interval training (hereon referred to as HIIT). HIIT sessions were conducted at the velocity equivalent to 95% $\dot{\text{V}}$O_2_peak, calculated via linear regression derived from the relationship between $\dot{\text{V}}$O_2_ and velocity. All sessions were supervised and established using the guidelines of Buchheit and Laursen (2013). Briefly, participants exercised for 2-min intervals followed by 2 min of static rest on the same treadmill used in previous sessions. Training frequency (3 sessions weekly) was separated by at least 24 h, with a mean rest of 52.8 ± 27.0 h and 2.2 ± 1.1 days between individual sessions. Exercise intensity remained constant throughout the intervention; however, the number of repetitions was incremental throughout the intervention (six in weeks 1, 2 and 6, eight in weeks 3 and 4 and ten in week 5).

Participants allocated to the MASK group completed whole-body HIIT training sessions whilst wearing an FRL mask (Phantom Training Mask, Phantom Athletics, Salzburg, Austria). The resistance was set at level 4 (of 4) for all participants irrespective of baseline *P*_Imax_. The mask was worn for the duration of each session, including during recovery periods providing an inspiratory resistance but no expiratory resistance. The IMT group completed HIIT but used a pressure threshold IMT device (POWERbreathe® classic series, HaB International, Warwickshire, UK) twice daily (morning and evening) throughout the intervention and the time between IMT and HIIT sessions was at least 1 h. Training load was set at 50% *P*_Imax_ and comprised thirty consecutive dynamic inspiratory efforts, twice daily, for six weeks. Each inspiratory effort was initiated from residual volume and participants endeavoured to maximise tidal volume (Faghy and Brown 2016). *P*_Imax_ was assessed bi-weekly during the training intervention, allowing the training load to be adjusted. CON completed HIIT only. Training compliance in all groups was monitored using a self-report training diary.

### Statistical analysis

Statistical analysis was performed using SPSS for Windows (SPSS, Chicago, IL, USA). A Shapiro–Wilk test was used to determine normality of the data. A repeated-measures, three-way ANOVA (independent variables: time, pre- and post-intervention across three groups) was used to assess changes over time in each time-trial, pre–post intervention and between each group. A Fishers Least Significant Difference (LSD) test was used to identify statistically different pre- to post-intervention changes. A priori α was set at 0.05, all results are presented as mean ± SD and effect size (ES) reported for pairwise comparisons. Effect sizes were calculated using Cohen’s *d* (*d* = (*x*^1^–*x*^2^)/pooled σ) and interpreted accordingly (small effect *d* = 0.2, moderate effect *d* = 0.5 and large effect *d* = 0.8).

## Results

Pre-intervention descriptive characteristics of participants are shown in Table 1 and there were no between-group differences in any variable (*p* > 0.05). Training compliance was high IMT (IMT 94 ± 8%) and in line with previous work in this area.

### Time-trial performance

**Fig. 2:**
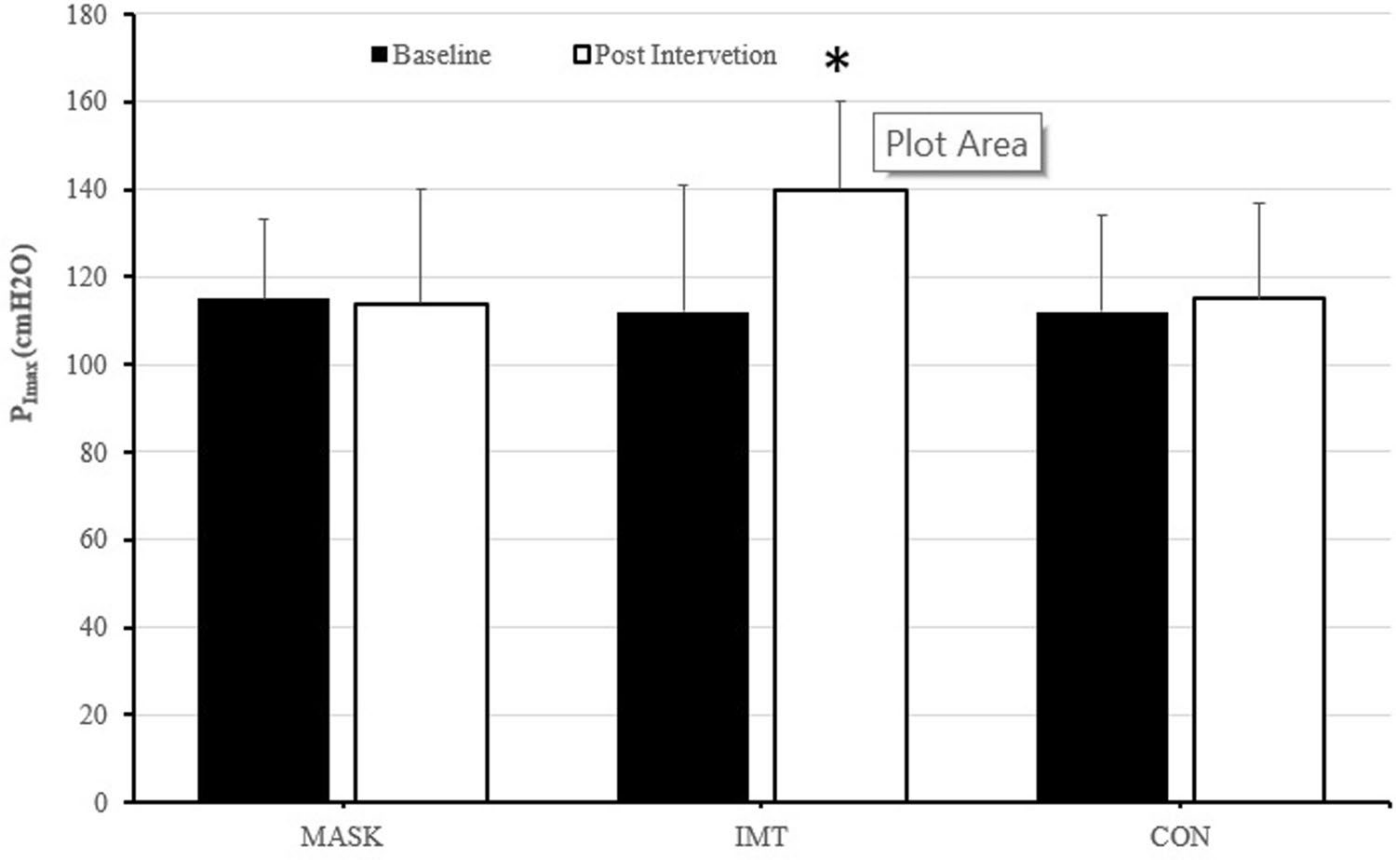
Performance on 5 km treadmill time-trial, closed bars represent pre-intervention trials and the open bars represent post-intervention performance. *Denotes a significant difference baseline, **a** denotes different from MASK and **b** denotes different from CON (*p* < 0.05)

Baseline, time-trial performance was not different between groups (MASK 26.14 ± 2.21 min, IMT 25.97 ± 2.07 min and CON 26.36 ± 2.42 min, *p* > 0.05). For time-trial performance, there was a main effect for pre–post intervention (*p* = 0.027, Fig. 2) and group (*p* = 0.018). Pre–post changes in time-trial performance were highlighted with a two-way interaction for group and time (*p* = 0.015). Time-trial performance was most improved in IMT (absolute reduction = 1.43 ± 0.48 min, 5.7 ± 1.5%, *p* = 0.007; effect size: *d* = 0.40). MASK (absolute reduction = 0.76 ± 0.33 min, 3.13 ± 1.7%, *p* = 0.034; effect size: *d* = 0.19) and CON (absolute reduction = 0.74 ± 0.47 min, 2.64 ± 1.0%, *p* = 0.043; effect size: *d* = 0.17) were also improved, but changes in performance were similar (*p* > 0.05).

### Respiratory function and diaphragm thickness

Pre-intervention values of *P*_Imax_ were not different between groups and unchanged following exercise (pooled mean pre: 124 ± 25 cmH_2_O vs post: 114 ± 21 cmH_2_O, absolute reduction 11 ± 13 cmH_2_O, 9 ± 10%, *p* = 0.358). Post-intervention *P*_Imax_ was improved in IMT only (pre: 112 ± 29 cmH_2_O vs post 140 ± 20 cmH_2_O, *p* = 0.008, absolute increase 34 ± 12 cmH_2_O, 32 ± 16%, effect size: d = 0.81). *P*_Imax_ was unchanged in MASK (pre: 115 ± 18 cmH_2_O vs post 114 ± 26 cmH_2_O, *p* = 0.445, effect size: *d* = 0.14) and CON (pre: 112 ± 31 cmH_2_O vs post 115 ± 22 cmH_2_O, *p* = 0.217, effect size: *d* = 0.25).

*T*_di_ was not different between groups at baseline (pooled mean 1.8 ± 0.3 mm) but was increased in IMT only (pre: 1.8 ± 0.2 mm vs post: 2.0 ± 0.2 mm, absolute change = 0.2 ± 0.2 mm, 9.5 ± 3.4%, *p* = 0.032, effect size *d* = 0.73) and was unchanged in MASK (pre: 1.9 ± 0.2 mm vs post: 1.9 ± 0.3 mm, absolute change = 0.1 ± 0.2 mm, *p* = 0.417, effect size *d* = 0.29) and CON (pre: 1.8 ± 0.3 mm vs post: 1.8 ± 0.2 mm, absolute change = 0.0 ± 0.2 mm, *p* = 0.283, effect size *d* = 0.12).

### Pulmonary function

Pre-intervention values of pulmonary function were not different between groups (Table 1) and were unchanged following both exercise and the intervention.

### Physiological and perceptual responses

Baseline and post-exercise values of HR were not different between groups (Tables 2, 3 and 4). All physiological and perceptual responses were similarly increased in each trial. La- was lower post exercise following the ISO trial post-intervention in all groups (see Tables 3, 4 and 5, *p* < 0.05). All other variables where unchanged.

**Table 3:** Physiological and perceptual responses for participants in the MASK group

	Pooled baseline	MASK (*n* = 8)					
		Pre-intervention TT		Post intervention (ISO-Time)		Post intervention TT	
		2.5 km	5 km	2.5 km	5 km	2.5 km	5 km
Time (min)	–	–	26.14 ± 2.21	–	26.14 ± 2.21	–	25.39 ± 2.28^B^
P_Emax_ (cmH_2_O)	110 ± 16 (88 ± 19%)	–	94 ± 18	–	98 ± 12	–	115 ± 11
La- (mmol·l^−1^)	1.6 ± 0.6	–	6.8 ± 3.4	–	5.0 ± 1.1^B^	–	6.4 ± 3.6^B^
HR (beats·min^−1^)	72 ± 14	160 ± 22	178 ± 13^a^	152 ± 17	170 ± 12^a^	166 ± 17	180 ± 13^a^
$\dot{\text{V}}$_E_ (L·min^−1^)	15.2 ± 5.4	92.2 ± 15.1	118.7 ± 28.5^a^	90.0 ± 22.1	112.2 ± 43.5^a^	96.5 ± 18.2	118.3 ± 31.2^a^
*V*_T_ (L)	0.9 ± 0.2	2.6 ± 0.5	2.7 ± 0.4	2.6 ± 0.8	2.8 ± 0.5^a^	2.3 ± 0.6	2.8 ± 0.5^a^
*f*_B_ (b min^−1^)	20 ± 6	39 ± 5	49 ± 4^a^	37 ± 6	44 ± 4^a^	40 ± 5	49 ± 4^a^
$\dot{\text{V}}$O_2_ (L·min^−1^)	0.54 ± 0.14	3.15 ± 0.67	3.51 ± 0.75^a^	3.03 ± 0.82	3.51 ± 1.11^a^	3.32 ± 0.82	3.78 ± 0.95^a^
$\dot{\text{V}}$CO_2_ (L·min^−1^)	0.47 ± 0.22	3.09 ± 0.66	3.55 ± 0.78^a^	2.98 ± 0.82	3.52 ± 1.38^a^	3.18 ± 0.85	3.91 ± 0.87^a^
RER	0.91 ± 0.06	0.98 ± 0.03	1.10 ± 0.12^a^	0.99 ± 0.08	1.13 ± 0.08^a^	0.96 ± 0.10	1.15 ± 0.08^a^
RPE (AU)	6 ± 0	11 ± 3	15 ± 0^a^	10 ± 3	14 ± 3^a^	11 ± 3	15 ± 2^a^
RPE_breathing_ (AU)	0 ± 0	3 ± 1	7 ± 2^a^	3 ± 1	5 ± 2^a^	3 ± 1	7 ± 2^a^

Values presented as mean ± SD. Maximum expiratory pressure (*P*_Emax_), values in parenthesis represent percentage of predicted, Heart rate (HR), Blood Lactate (La-), minute ventilation (*V*_E_), tidal volume (*V*_T_), breathing frequency (*f*_B_), oxygen consumption ($\dot{\text{V}}$O_2_), carbon dioxide production $\dot{\text{(V}}$CO_2_), Respiratory exchange ratio (RER), Rating of Perceived Exertion (RPE), Rating of Perceived Exertion For Breathing (RPE_breathing_), Arbitrary units (AU). A denotes change between 2.5 km and 5 km values and B represents the difference between trials (*p* < 0.05)

**Table 4:** Physiological and perceptual responses for participants in the CON group

	Pooled baseline	CON (n = 7)					
		Pre-intervention TT		Post Intervention (ISO-Time)		Post Intervention TT	
		2.5 km	5 km	2.5 km	5 km	2.5 km	5 km
Time (min)	–	–	26.36 ± 2.42	–	26.36 ± 2.42	–	25.62 ± 1.70^B^
P_Emax_ (cmH_2_O)	113 ± 14 (103 ± 22%)	–	103 ± 18	–	105 ± 17	–	109 ± 20
La- (mmol·l^−1^)	1.6 ± 0.6	–	8.8 ± 2.7	–	5.9 ± 2.6^B^	–	8.2 ± 2.3
HR (beats·min^−1^)	87 ± 18	155 ± 12	179 ± 12^a^	160 ± 14	181 ± 16^a^	168 ± 5	186 ± 4^a^
$\dot{\text{V}}$_E_ (L·min^−1^)	14.9 ± 4.9	70.2 ± 20.0	82.5 ± 22.9	74.6 ± 16.0	83.5 ± 17.9	81.8 ± 16.9	91.3 ± 15.5^a^
*V*_T_ (L)	0.9 ± 0.3	2.3 ± 0.6	2.3 ± 0.6	2.2 ± 0.4	2.4 ± 0.7	2.4 ± 0.5	2.6 ± 0.5
*f*_B_ (b min^−1^)	16 ± 6	34 ± 7	44 ± 8^a^	31 ± 6	40 ± 6^a^	36 ± 8	46 ± 5^a^
$\dot{\text{V}}$O_2_ (L·min^−1^)	0.48 ± 0.20	2.66 ± 0.48	2.90 ± 0.39	2.54 ± 0.44	2.87 ± 0.60	2.70 ± 0.44	3.12 ± 0.41
$\dot{\text{V}}$CO_2_ (L·min^−1^)	0.39 ± 0.18	2.47 ± 0.80	2.79 ± 0.89	2.53 ± 0.49	2.95 ± 0.61	2.76 ± 0.54	3.21 ± 0.49
RER	0.91 ± 0.06	1.01 ± 0.04	1.06 ± 0.10	1.00 ± 0.08	1.13 ± 0.07	1.02 ± 0.05	1.13 ± 0.03
RPE (AU)	6 ± 0	12 ± 3	15 ± 2^a^	12 ± 1	14 ± 1^a^	12 ± 1	16 ± 1^a^
RPE_breathing_ (AU)	0 ± 0	3 ± 1	5 ± 2^a^	3 ± 1	6 ± 2^a^	3 ± 1	7 ± 1^a^

Values presented as mean ± SD. Maximum expiratory pressure (*P*_Emax_), values in parenthesis represent percentage of predicted, Heart rate (HR), Blood Lactate (La-), minute ventilation (*V*_E_), tidal volume (*V*_T_), breathing frequency (*f*_B_), oxygen consumption ($\dot{\text{V}}$O_2_), carbon dioxide production $\dot{\text{(V}}$CO_2_), Respiratory exchange ratio (RER), Rating of Perceived Exertion (RPE), Rating of Perceived Exertion For Breathing (RPE_breathing_), Arbitrary units (AU). A denotes change between 2.5 km and 5 km values and B represents the difference between trials (*p* < 0.05)

## Discussion

This study aimed to determine whether a flow-resistive device worn during exercise led to increased time-trial performance above the benefits to performance provided by HIIT. The key finding demonstrates that flow-resistive masks worn during HIIT training provided no additional benefit to 5 km running performance when compared to HIIT training only. Second, all interventions improved 5 km running performance but the relative improvement in IMT was greater than the improvement observed in MASK and CON. No change in physiological and perceptual responses with masks was observed when compared to baseline, post-intervention ISO trial and post-intervention all-out trial.

Typically, respiratory muscle training techniques are conducted as an adjunct to exercise training regimes. The use of face masks during exercise poses an inspiratory resistive load that is proportional to inspiratory flow rates observed during exercise, therefore increasing the work of breathing. However, the effectiveness and supporting datasets for these devices are limited and have not been compared against other IMT methods. Porcari et al. (2016), incorporated the Elevation Training Mask 2.0 into 6 weeks of high-intensity cycle ergometer training and reported increased endurance performance (power output at ventilatory threshold) but there were no changes in maximal exercise performance ($\dot{\text{V}}$O_2max_ or peak power output) when expressed relative to the improvement observed in the control group. This may be expected as it is well documented that $\dot{\text{V}}$O_2max_ is unaffected by IMT methods since it fails to influence any point of the physiological determinants that comprise $\dot{\text{V}}$O_2max_ (Brown et al. 2010)_._ More recently Segizbaeva and Aleksandrova (2018) demonstrate improved respiratory muscle strength and endurance after 12 weeks of using an FRL face mask during running exercise and improved 100 m and 3000 m running performance (3% and 7%, respectively). Whilst the mechanism of improved performance is not explored by the authors, it is likely the result of acclereated O_2_ kinteics at the onset of exercise, as previously demonstrated by our group (Brown et al. 2012). It is, however, unclear how 100 m sprint performance would be improved following FRL training as the combined time and exercise intensity is unlikely to result in a significant contribution from ventilatory parameters (Harms et al. 2000). The onset of respiratory muscle fatigue, the respiratory muscle metaboreflex and associated sensations of respiratory and/or locomotor discomfort are also unlikely when considering the known determinants of respiratory muscle work that influence performance (Dempsey et al. 2006). Furthermore, Jagim et al. (2018) report that wearing an FRL mask during resistance exercise does not impede the ability to complete resistance training sessions. The authors noted acute increases in metabolic stress markers (La-), ratings of mental fatigue and a reduction in the peak velocity during inspiratory efforts measured during resistance activities (back squat and bench press). The authors did report concerning side effects, which included reduced alertness and focus during resistance exercises, the underlying mechanism causing this change was not determined and increased mechanistic understanding is needed here.

The results of this current study demonstrate that the mask which mimics FRL yields no additional benefit to treadmill running performance when used in conjunction with HIIT training. As previously mentioned, FRL specifically loads the inspiratory muscles using either high-force/low-velocity or low-force/high-velocity contractions. A determinant of this methods is the need to maintain a sufficient load to the inspiratory muscles, according to Ohms law of resistance. Although previous work demonstrates that HIIT produces high flow rates and leads to increased *P*_Imax_ (Dunham and Harms 2012), these improvements were not observed in the current study. This is likely due to the absence of high flow rates providing an insufficient stimulus for the respiratory muscles to adapt, as demonstrated in previous research adopting FRL techniques where ventilation is controlled (Mickleborough et al. 2010). Recent data support this notion and emphasise the need for sufficiently strenuous, inspiratory flow-resistive loads (> 50% *P*_Imax_) to elicit an adaptive response and whilst this did not influence peak transdiaphragmatic pressure, markers of oxidative stress were elevated (Briskey et al. 2020). Although not measured as part of this study, the resistance posed by the masks during exercise is an important consideration in their overall effectiveness and future research should look to quantify the inspiratory load and the work of breathing during exercise with a flow-resistive load mask to determine whether these methods provide a sufficient and consistent overload stimulus that could lead to chronic adaptations within the respiratory muscles.

The present study demonstrates that pressure threshold IMT that is conducted as an adjunct to exercise training continues to be an effective ergogenic aid for sports performance. The results here demonstrate a 5.7 ± 1.5% (mean reduction 1.43 ± 0.48 min) increase in 5 km TT performance which is greater than the improvement in HIIT alone 2.7 ± 1.0% (mean reduction = 0.74 ± 0.47 min). It is important here to acknowledge that the absence of a placebo group and that further research is needed to confirm true ergogenic properties of IMT. PTL methods use progressive overload to the respiratory muscles which results in beneficial adaptations and increases exercise performance (for a full review see (HajGhanbari et al. 2013). Our findings are consistent with previous data that demonstrate improved performance in a range of exercise modalities which include but is not limited to, middle distance and endurance running (Ross et al. 2008; Edwards et al. 2008), cycling (Romer et al. 2002; Gething et al. 2004) (including during hypoxic conditions (Salazar-Martínez et al. 2017), swimming (Shei et al. 2016), rowing (Griffiths and McConnell 2007) and during occupational performance tests (Faghy and Brown 2014; Shei et al. 2018). The mechanism by which IMT improved performance is in line with previous work in the area which demonstrates increased *P*_Imax_ and *T*_di_. Chronic interventions (typically 4–6 weeks) repeatedly demonstrate neural adaptations (Hawkes et al. 2007) and structural changes (Enright et al. 2006; Downey et al. 2007) which augment respiratory muscle strength, endurance and efficiency and during exercise allows the respiratory muscles to work at a lower relative intensity during exercise.

Despite attempts to improve the ecological validity of FRL methods, the findings of this study demonstrate that these masks that are worn during exercise training yield no additional benefit to running performance above those posed by HIIT training. This is likely, although yet to be confirmed due to insufficient stimuli that are presented to the inspiratory muscles during exercise tasks, thus resulting in no adaptation to respiratory muscle strength and/or endurance. The use of PTL IMT techniques that are used as an adjunct to training demonstrate improved respiratory strength, endurance and exercise performance and should be used in favour of methods that seek to combine exercise and IMT.

## Conclusion

IMT used as an adjunct to training improves running performance above the benefits provided by HIIT training. The use of a flow-resistive training mask worn during HIIT sessions yields no additional benefit to HIIT alone. We suggest that this is due to insufficient inspiratory load and further research should seek to quantify the work of breathing and flow rates during exercise which are known determinants of the adaptions associated with flow-resistive loading methods.

## Abbreviations

$\dot{\text{V}}$CO_2_: Carbon dioxide production
$$\dot{\text{V}}$$_E_: Minute ventilation
$$\dot{\text{V}}$$O_2_: Oxygen consumption
$\dot{\text{V}}$sO_2_peak: Peak oxygen uptake
ANOVA: Analysis of variance
CON: Control
FEV_1_: Forced expired volume in one second
FRL: Flow resistive loading
FVC: Forced vital capacity
HIIT: High-intensity interval training
HR: Heart rate
IMT: Inspiratory muscle training
La: Blood lactate
PEF: Peak expiratory flow
*P*_Emax_: Maximal expiratory pressure
*P*_Imax_: Maximal inspiratory pressure
RER: Respiratory exchange ratio
*T*_di_: Diaphragm thickness
